# Eribulin induces micronuclei and enhances the nuclear localization of cGAS in triple-negative breast cancer cells

**DOI:** 10.21203/rs.3.rs-3672056/v1

**Published:** 2023-12-06

**Authors:** Hideyuki Yamada, Mamoru Takada, Dhaval Ghone, Muhan Yu, Takeshi Nagashima, Hiroshi Fujimoto, Junta Sakakibara, Yoshie Hasegawa, Shintaro Takao, Akimitsu Yamada, Kazutaka Narui, Takashi Ishikawa, Aussie Suzuki, Masayuki Otsuka

**Affiliations:** Chiba University; Chiba University; University of Wisconsin–Madison; Chiba University; Chiba University; Chiba University; Chiba University; Hachinohe City Hospital; Konan Medical Center; Yokohama City University; Yokohama City University Medical Center; Tokyo Medical University; University of Wisconsin–Madison; Chiba University

## Abstract

Eribulin (ERI), clinically utilized for locally advanced or metastatic breast tumors, has shown potential links to the immune system. Notably, the cGAS-STING pathway, a key component of innate immunity, has gained prominence. Yet, limited reports explore ERI’s effects on the cGAS-STING pathway. Additionally, the nuclear presence of cGAS remains poorly understood. This study uniquely delves into ERI’s impact on both the cytosolic cGAS-STING pathway and nuclear cGAS. ERI enhances nuclear localization of cGAS, resulting in hyper-activation of the cGAS-STING pathway in triple-negative breast cancer cells. Reduction of cGAS heightened both cell proliferation and ERI sensitivity. In clinical data using ERI in a neo-adjuvant setting, patients with low cGAS cases exhibited reduced likelihood of achieving pathological complete response after ERI treatment. These findings illuminate the potential of cGAS and IFNβ as predictive biomarkers for ERI sensitivity, providing valuable insights for personalized breast cancer treatment strategies.

## Introduction

Eribulin (ERI) is one of the microtubule-targeting agents (MTAs) and is known as a microtubule polymerization inhibitor.^1^ Our study assumes pivotal significance in unraveling the distinct anti-tumoral efficacy of ERI, a drug predominantly employed in the context of metastatic recurrent breast cancer and for tumors that have developed resistance to other MTAs, exemplified by Paclitaxel (PTX). The inherent challenge in clinical settings lies in ascertaining the sensitivity of ERI when administered in preoperative chemotherapy. In this context, our analysis, drawing from samples collected in the preoperative chemotherapy study of ERI (JONIE-3 study), stands as a unique opportunity.^2^ This invaluable dataset allows us to dissect and comprehend the unadulterated anti-tumoral impact of ERI in breast cancer—a crucial endeavor that can significantly advance our understanding and inform clinical strategies. The outcomes of this analysis hold the potential to reshape therapeutic paradigms and refine the clinical application of ERI in breast cancer management.

Chromosomal instability (CIN) is a phenomenon in which chromosome missegregation persists overconsecutive cell division.^3^ Triple negative breast cancers (TNBCs) often exhibit abnormal cell division and increased CIN with PTX treatment.^4^ However, the relationship between ERI and CIN has rarely been reported. Cells with mitotic defects during chromosome segregation often have micronuclei. Recent studies indicate that micronuclei, particularly nuclear membrane raptured micronuclei, can activate the cyclic GMP-AMP synthase (cGAS) pathway.^5^ The multifaceted roles of the cGAS pathway are recognized innate immune sensor. The cGAS pathway is acknowledged for its surveillance of the cytosol, detecting microbial DNA, and responding to self-DNA, either from the nucleus during genomic stress or from stressed mitochondria.^6–9^ Activation of cGAS occurs upon recognition of double-stranded DNA, catalyzing the formation of cyclic GMP-AMP (cGAMP).^10–14^ The ensuing binding of cGAMP to its adaptor STING triggers downstream innate immune responses.^15^ Dysregulation of the cGAS-STING pathway is implicated in various disorders, spanning infections, inflammatory diseases, neurodegeneration, and cancer.^16^ While cGAS is traditionally considered a cytosolic protein, it undergoes dynamic changes in subcellular localization.^17^ It transiently accumulates in the nucleus during mitotic nuclear membrane dissolution and actively translocates into the nucleus in response to DNA damage.^18^ Intriguingly, cGAS has also been reported at the plasma membrane in certain cell types.^19^ Despite these insights, ongoing debates persist regarding the subcellular localization and functions of cGAS across diverse biological conditions.^20^ This paper aims to contribute to this discourse, shedding light on the nuanced roles of cGAS in different cellular contexts.

## Results

### ERI but not PTX inhibited microtubule polymerization.

In our investigation of ERI’s impact on cell division, we employed a tubulin polymerization assay to assess its effects, given its role as a microtubule polymerization inhibitor, similar to PTX. Confirming earlier reports, ERI exhibited a dose-dependent suppression of microtubule polymerization, effectively inhibiting this process at a concentration as low as 10 nM. Conversely, PTX, acting as a microtubule stabilizer, did not inhibit microtubule polymerization. **(Supplementary Fig. 1).**

### ERI induced distinct mitotic abnormalities compared to those caused by PTX.

Recent studies have shown that the concentration of PTX in breast cancer tumors tends to be lower than previously expected.^21^ In line with these findings, an in vitro PTX concentration of ~ 10 nM has been estimated as the clinically relevant concentration. Consequently, we utilized a 10 nM concentration for both ERI and PTX in our live cell imaging experiments.

**Figure 1:**
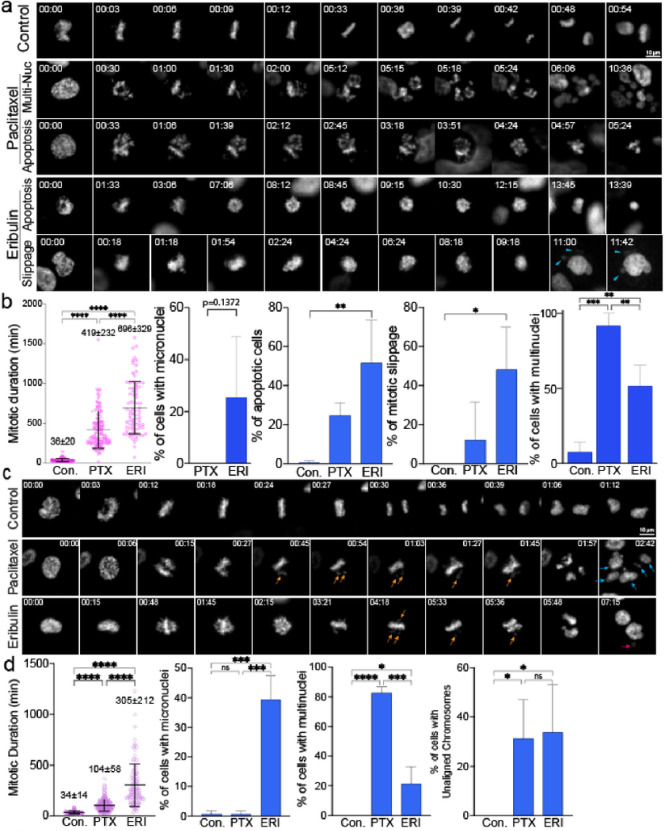
Live cell imaging was used to show the effects of ERI on cell division. (a) MM231 cells and (c) RPE1 cells were treated with DMSO or PTX or ERI. Representative images of the mitotic morphology for the cells are shown. For (b) MM231 cells and (d) RPE1 cells, the length of mitotic duration and the ratio of cells with characteristic mitotic abnormalities are shown.

Initially, we conducted high-temporal live cell imaging to observe the differential effects of ERI and PTX during mitosis. Our findings revealed that both ERI and PTX at their clinical concentrations caused prolonged mitosis in MDA-MB-231 (MM231) cell lines. Notably, 10 nM of ERI led to a significantly longer duration of mitotic arrest, lasting about 696 minutes, compared to 419 minutes with 10 nM of PTX (Fig. 1a, 1b). Moreover, we found that ERI uniquely triggered the formation of micronuclei, a phenomenon not seen with PTX. Both agents increased the presence of multinucleated cells, with PTX showing a higher propensity to induce this effect than ERI.

While it is unclear why ERI tends to induce micronuclei and PTX leads to multinuclear formation, these effects are likely due to a higher frequency of unaligned chromosomes and mitotic slippage, which are both major mitotic defects.^22^ Consistent with the MM231 results, both PTX and ERI induced prolonged mitosis in Retinal Pigment Epithelium cell lines (RPE1) cells; in particular, ERI significantly prolonged mitosis more than PTX (Fig. 1c, 1d). In summary, while both ERI and PTX at clinical concentrations lead to extended mitosis and chromosome misalignment, ERI notably causes more severe mitotic arrest and a higher incidence of micronuclei.

We further investigated the distinct impact of ERI and PTX on CIN using MM231 and RPE1 cells. For MM231, we established five cell lines subjected to various treatments: DMSO (MM231-DMSO), short-term paclitaxel (PTX-short: 1 μM for 24 hours), long-term paclitaxel (PTX-long: 1 nM for 60 days), short-term eribulin (ERI-short: 10 nM for 24 hours) and long-term eribulin (ERI-long: 0.2 nM for 60 days). We assessed both acute effects (24 hours), simulating one cell cycle, and chronic effects (2 months), mimicking clinical resistance to these drug treatments. In the case of RPE1, three cell lines were established: DMSO-treated (RPE-DMSO), short-term paclitaxel (PTX-short: 1 μM) and short-term eribulin (ERI-short: 10 nM). Unfortunately, long-term cultivation of drug-treated RPE1 cells were impractical due to induced cell death.

**Figure 2:**
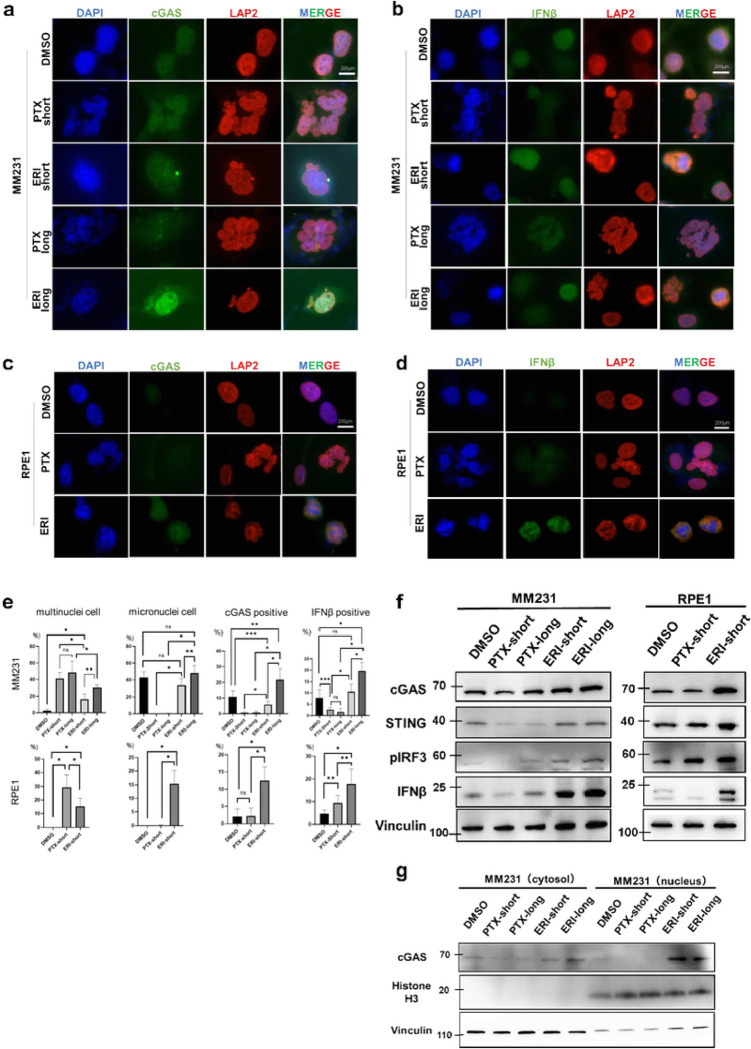
The effects of ERI on cGAS was evaluated. Immunofluorescence with (a) cGAS or (b) IFNβ was performed with MM231 cells treated with DMSO or PTX-short or PTX-long or ERI-short or ERI-long. Identically, Immunofluorescence with (a) cGAS or (b) IFNβ was performed with RPE1 cells treated with DMSO or PTX-short or PTX-long. DAPI and LAP2 were used for nuclear staining. (e) The number of characteristic cells was counted and statistically compared. The meaning of the asterisks are as follows: * p<0.001, **:p<0.01, *** p<0.05 (f) MM231 cells and RPE1 cells were used and their protein expression of cGAS, STING, pIRF3 and IFNβ was evaluated by western blotting. Vinculin was used as loading control. (g) The cGAS expression of the cytoplasmic and nuclear fractions was evaluated by cell fractionation assay. Vinculin was used as cytoplasmic loading control and Histone H3 as nuclear loading control.

Recent studies have demonstrated that micronuclei, rather than multinuclei, are capable of activating the cGAS-STING pathway.^23^ We posited that treatment with ERI might induce a more pronounced activation of the cGAS-STING pathway compared to PTX, given ERI’s propensity to preferentially induce micronuclei. To evaluate this hypothesis, we conducted quantitative immunofluorescence analyses of cGAS and IFNβ in the above established cell lines. Our findings revealed that ERI treatment resulted in an upregulation of cGAS and IFNβ expression, with a notable increase in cGAS expression in cells treated with ERI over a longer duration compared to a shorter one. Remarkably, ERI treatment led to heightened cGAS expression and its nuclear accumulation (Fig. 2a, 2b) Consistent with the results observed in MM231 cells, ERI also increased the expression of both cGAS and IFNβ in RPE1 cells, suggesting a conserved effect. (Fig. 2c, 2d).

We quantified the frequency of cells positive for cGAS, IFNβ, multinucleation, and micronuclei in MM231 and RPE1 cells treated with short and long durations of PTX or ERI (Fig. 2e). In both cell types, PTX treatment resulted in a significantly higher frequency of multinucleated cells compared to ERI treatment. Furthermore, ERI treatment uniquely increased the presence of micronuclei in MM231 and RPE1 cells relative to PTX treatment. These observations align with those from high-temporal live cell imaging studies (Fig. 1a, 1c). Notably, ERI treatment significantly enhanced the frequency of cGAS-, and IFNβ-positive cells compared to PTX in both MM231 and RPE1 cells. In MM231 cells, prolonged ERI treatment further augmented the incidence of micronuclei, cGAS-and IFNβ-positive cells compared to shorter treatment durations, suggesting that longer exposures to lower concentrations of ERI, more akin to clinical conditions, may further activate the cGAS pathway. These results highlight the distinct mitotic defects induced by ERI and PTX, with ERI uniquely stimulating the cGAS pathway.

### ERI upregulates cGAS expression and accumulates in the nucleus

To determine if ERI treatment upregulates overall cGAS expression, we performed western blotting (WB) to measure cGAS protein levels in PTX-or ERI-treated cells (Fig. 2f). In line with quantitative immunofluorescence (IF) results, both MM231 and RPE1 cells exposed to ERI showed increased expression of cGAS and STING, a crucial cGAS binding partner, compared to control and PTX-treated cells. Additionally, phospho-IRF3 and IFNβ, activators downstream of the cGAS-STING pathway, were elevated in ERI-treated cells. Extended exposure to ERI (ERI-long) further enhanced cGAS-STING and its downstream activators, suggesting that prolonged ERI exposure intensifies the activation of the cGAS-STING pathway. Observing enhanced nuclear accumulation of cGAS in ERI-treated cells (Fig. 2a), we assessed the cGAS protein levels in nuclear versus cytoplasmic fractions. To do this, we extracted proteins from the MM231 cell line, dividing them into cytoplasmic and nuclear fractions (Fig. 2g). We found that ERI treatment not only increased cGAS levels in the cytoplasmic fraction but also in the nuclear fraction, aligning with the quantitative IF results, compared to control or PTX-treated cells. Overall, these findings suggest that cells treated with ERI exhibit heightened activation of the cGAS-STING pathway relative to control or PTX-treated cells.

### Knockdown-cGAS accelerates proliferation of triple negative breast cancer cells.

**Figure 3:**
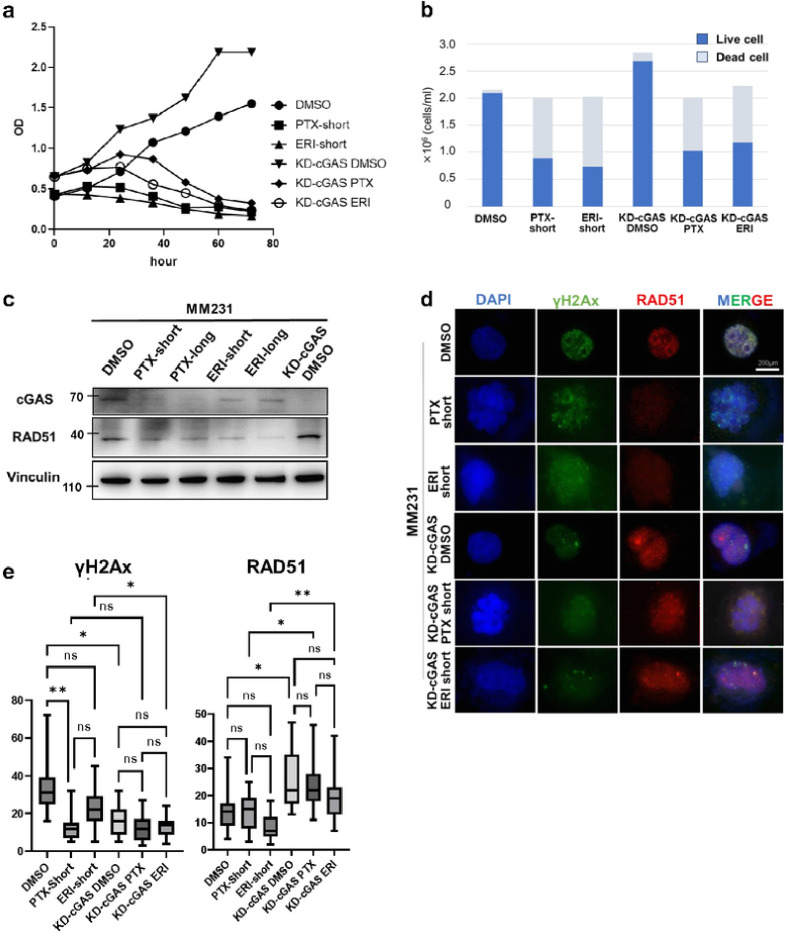
The effects of KD-cGAS on cell proliferation was evaluated. MM231 cells (DMSO, PTX-short, ERI-short, KD-cGAS-DMSO, KD-cGAS-PTX, KD-cGAS-ERI) were used (a) Proliferation assay was performed. 24 hours after knockdown of cGAS was defined as 0h, and cell proliferation was evaluated every 12 hours. (b) Tripan blue stain was used to evaluate the percentage of live and dead cells 24 hours after treatment. (c) We evaluated the effects of knocking down of cGAS on RAD51 expression. Vinculin was used as loading control. (d) Immunofluorescence with RAD51 and γH2Ax was performed. DAPI was used for nuclear staining. (e) We counted the number of nuclear foci in each cell stained with γH2Ax and RAD51. The meaning of the asterisks are as follows: * p<0.05, ** p<0.01.

We examined the effects of knockdown-cGAS (KD-cGAS) on cell growth with the combination of PTX or ERI treatments. To this, we first evaluated the knockdown efficiency of four different siRNAs (siRNA5, siRNA6, siRNA7 and siRNA8) of cGAS (**Supplementary Fig. 2a**), confirming that cGAS was knocked down in all four siRNAs by WB and RT-PCR, with a knockdown efficiency of approximately 70% (**Supplementary Fig. 2b**). While cGAS knockdown did not alter the cell proliferation as compared to control, KD-cGAS or control cells treated with ERI or PTX declined cell growth. KD-cGAS or control cells treated with ERI or PTX declined cell growth (Fig. 3a). To further determine cell survival, we counted live and dead cells at 24 hours after PTX or ERI treatment in control and KD-cGAS cells. We found that KD-cGAS increased the number of viable cells. Furthermore, KD-cGAS treated with ERI slightly increased the ratio of viable cells compared to ERI-short, while PTX did not alter the ratio of viable cells to dead cells much. (Fig. 3b). In IF of KD-cGAS cells, cGAS staining was reduced compared to non-KD-cGAS, but no difference in mitotic morphology was observed between KD-cGAS and non-KD-cGAS cells **(Supplementary Fig. 2c**).

It has been reported that both cGAS-STING pathway and the DNA damage response are tightly linked.^23–26^ We then assessed RAD51 protein expression to examine the nuclear effects of cGAS. KD-cGAS upregulated RAD51 expression compared to non-KD-cGAS (Fig. 3c). DNA damages are known to induce cGAS translocation into the nucleus.^5^ To test whether ERI-treated cells induce DNA damages, we stained γH2AX and RAD51, which are specifically accumulated at the DNA damage cites. Interestingly, compared to DMSO we found that KD-cGAS-DMSO seemed to increase the accumulation of RAD51 and reduced γH2AX in the nucleus. Compared to ERI short, KD-cGAS-ERI also seemed to increase the accumulation of RAD51 and decreased γH2AX in the nucleus (Fig. 3d). Then we focused on the foci formation of RAD51 and γH2Ax in the nucleus. KD-cGAS-DMSO significantly increased RAD51 foci and decreased γH2Ax foci predominantly compared to non-KD-cGAS-DMSO. Moreover, KD-cGAS-ERI increased RAD51 foci and decreased γH2Ax foci compared to ERI-short (Fig. 3e). These results indicate that foci formation of RAD51 may be partially involved in the cGAS pathway in breast cancer cells.

### Patients with low cGAS and high RAD51 associated with non pCR after chemotherapy

**Figure 4:**
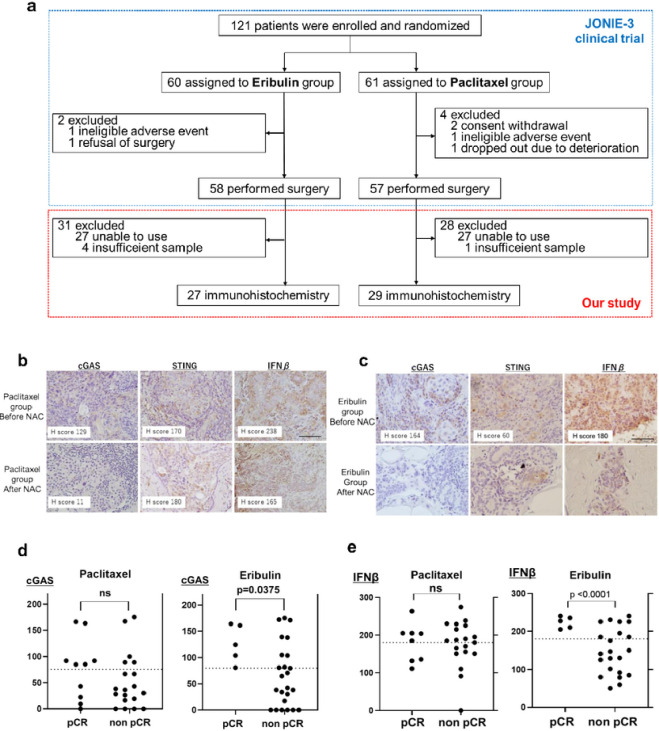
Immunostaining was performed with clinical samples. (a) The method of the patients-extraction is shown. (b) In PTX group and (c) In ERI group, representative immunostaining images before and after chemotherapy are stained with cGAS, STING and IFNβ. (d) The H-score of cGAS was plotted for both PCR and non-PCR cases in both ERI and PTX group. (e) Similary, the H-score of IFNβ was plotted.

In order to assess how cGAS expression levels influences the effects of ERI in clinical outcomes, we first performed immunohistochemistry (IHC) of 56 biopsy specimens obtained by breast cancer patients who were enrolled the JONIE-3 clinical trial for neoadjuvant setting.^2^ This study compared ERI treatment group with PTX treatment group for 12 weeks, followed by 4 cycles of fluorouracil, epirubicin and cyclophosphamide (FEC). The pathological complete response (pCR) rates were 20.7% in the ERI group and 29.8% in the PTX group, and there was no significant difference between them. The method of the patients-extraction is shown in Fig. 4a.

We examined if cGAS, STING and IFNβ expression were associated with pCR in the PTX and ERI groups. Representative IHC images with each expression score of cGAS, STING and IFNβ are shown in Fig. 4b (ERI) and 4c (PTX). The intensity of the staining was evaluated by Histoscore (H-score) at hot spot.^27^ cGAS (H-score: 0–175) and IFNβ (H-score: 0–274) were predominantly localized in both the cytoplasm and nucleus; STING (H-score: 0–220) was stained in the cytoplasm and RAD51 (H-score: 0–270) was stained in the nucleus.

We quantified and compared cGAS and IFNβ levels between pCR cases and non pCR cases (Fig. 4d). We found that the expression of low cGAS was significantly correlated with non pCR (p = 0.0375) in the ERI group, while the correlation between cGAS and pCR was not observed in the PTX group (p = 0.2983) (Fig. 4e). Similar to cGAS profiles, low IFNβ also correlated with non pCR (p < 0.0001) in the ERI group, while there was no significant correlation between IFNβ and pCR (p = 0.2983) in the PTX group. Furthermore, cGAS and IFNβ showed a moderate positive correlation in the ERI group (R = 0.4690) but not in the PTX group (R = 0.0140) (**Supplementary Fig. 3, Supplementary Table 1**). Notably, in the ERI group, ~ 50% of the patients with high cGAS and IFNβ achieved pCR. Interestingly, in both ERI and PTX groups, STING was not significantly correlated with pCR (Table. 1a). These results suggest that a sensitivity to ERI was decreased in the cells with low cGAS or IFNβ in a treatment-naïve breast cancer. On the other hand, in the ERI group, high RAD51 tended to be related to non pCR, but the difference was not statistically significant (p = 0.1233). Especially, high cGAS and low RAD51 cases are more likely to achieve pCR compared to the others (Table. 1b).

## Discussion

TNBCs have the strongest tumor immunogenicity among all breast cancer subtypes, and immunotherapies targeting PD-1 and PD-L1 have shown efficacy, ^28^ but therapies targeting cGas/STING are not yet practical and the efficacy of TNBCs as therapeutic targets is still unclear. cGAS, a known sensor of foreign DNA in pathogens and tumors, and known to activate type I IFNs by STING, has recently attracted attention as a good target for cancer therapy.^7^ The present study reveals a new aspect of ERIs, which are microtubule polymerization inhibitors, but their mechanism for anti-tumor activity is not fully understood.

DNA damage can result from exposure to drugs or radiation, leading to various abnormal cell division processes. Conversely, recent studies have indicated that mitotic defects themselves may be a source of DNA damage. Major mitotic defects, such as misaligned chromosomes, lagging chromosomes and chromosomal bridges, can lead to the formation of micronuclei.^5,29^ While both ERI and PTX disrupt proper microtubule dynamic instability,^30^ our findings show that ERI treatment predominantly induces micronuclei, whereas PTX treatment results in multinuclei. This distinction is critical, as micronuclei are known to trigger cGAS-STING activation. Consequently, ERI has the potential to enhance the effectiveness of immune checkpoint inhibitors.

Although cGAS is also thought to localize to the nucleus and binds to chromatin,^31^ and there are a few reports on the role of nuclear cGAS. The following roles for nuclear cGAS have been reported: regulation of innate immune responses,^32,33^ suppression of homologous recombination.^24^ Another previous study reported that knockdown of cGAS inhibited tumor growth through stabilization of the replication forks in lung cancer cells.^25^ On the other hand, it has also been reported that the promotion of DNA repair might accelerate cell proliferation, and furthermore, RAD51 inhibitor enhanced sensitivity to radiation or drugs.^34,35,36^ Our results indicate that ERIs accumulate nuclear cGAS and increase their own sensitivity through delayed DNA damage repair. PTX and ERI are both microtubule inhibitors, but have different detailed mechanisms. It is interesting to note how these mechanistic differences can have a profound impact on the effects they have on cancer cells, such as CIN and inhibition of DNA repair.

It was highly significant that immunostaining was performed with a sample of a drug-naïve tumor before the effects of ERI. Because clinical indication of eribulin is later line after failure of microtubule inhibitors in metastatic breast cancer, and it is impossible to determine the effect of pure eribulin in a normal clinical specimen. We also considered evaluating if the cGAS-STING pathway is associated with overall survival and recurrence-free survival. However, due to the small number of cases (Fig. 4a), the relapses (2 in total) and the lack of prognostic follow-up period in the JONIE-3 trial, this could not be evaluated in the present study. Focusing on the pCR rate, which is known to be related to prognosis used instead of prognostic analysis, cases with low cGAS, low IFNβ and high RAD51 were less able to achieve pCR. Considering these clinical data together with Fig. 3b, nuclear cGAS which inhibits RAD51 may increase susceptibility to ERI and may be a biomarker to infer ERI efficacy.

In the present report, ERI activated the cGAS-STING pathway more than PTX, similar to previous reports.^37,38^ We considered that it may be clinically important to note that ERI in particular accumulates cGAS in the nucleus and subsequently delays DNA damage repair pathways. Hence, the present study has newly identified the possible role of ERIs in a continuous positive loop, whereby ERI increases DNA damage, leading to increased nuclear cGAS accumulation, which then inhibits homologous recombination and subsequently further increases DNA damage. Since strategies targeting DNA repair pathways, such as PARP inhibitors, are still extremely important as a treatment strategy for TNBC, and are expected to be further developed in the future, our data seems to be an important study for future breast cancer treatment.

## Materials and Methods

All methods were performed in accordance with the relevant guidelines and regulations.

### Cell culture.

MDA-MB-231 (MM231) and retinal pigment epithelium (RPE1) cells were cultured in Dulbecco’s Modified Eagle Medium (DMEM) containing 10% fetal bovine serum (FBS) and 1% Penicillin-Streptomycin. Five types of cells were derived from MM231. Derived cells were MM231 with DMSO (MM231-DMSO), MM231 with paclitaxel for short term (PTX-short) (1μM), MM231 with paclitaxel for long term (PTX-long) (1 nM), MM231 with eribulin for short term (ERI-short) (10 nM), MM231 with eribulin for long term (ERI-long) (0.2 nM). Three types of cells were derived from RPE. Derived cells were RPE with DMSO (RPE-DMSO), RPE with paclitaxel for short term (PTX-short) (1 μM), RPE1 with eribulin for short term (RPE-ERI) (10 nM). PTX-short and ERI-short were incubated for 24hours, while PTX-long and ERI-long were incubated for 60days. All cells were maintained at 37°C in a 5% CO2 incubator. ERI and PTX were dissolved in DMSO. ERI was stored at −80°C, PTX was stored at −20°C.

### Reagents and antibody.

ERI was from Fuji Film and PTX was from ADIPOGEN. The following antibodies were used for WB: cGAS (#15102S, 1:1000), STING (#13647, 1:1000), pIRF3 (#4947, 1:1000), Histone H3 (#9715, 1:1000), RAD51 (#D4B10, 1:1000), anti-rabbit IgG (#7074, 1:2000) and anti-mouse IgG (#7076, 1:2000) were from Cell Signaling Technology (CST), IFNβ (#PA5–20390, 1:1000) was from Invitrogen and Vinculin was from Santa cruz (#A1121, 1:1000). The following antibodies were used for IHC: STING (#13647, 1:400) was from Cell Signaling Technology and IFNβ (#PA5–20390, 1:400) was from Invitrogen. The following antibodies were used for IF: cGAS (#79978S, 1:500) was from CST, RAD51 (#ab133534, 1:500) was from abcam. IFNβ (#PA5–20390, 1:400) was from Invitrogen and LAP2 (#8197900, 1:1000) was from BD Biosciences. DNA Damage Detection Kit (#340–09431) was from Fuji Film.

### Tubulin Polymelization Assay.

The tubulin polymerization experiment was performed as per the reported protocol described in the assay kit (#BK006P). Tubulin protein (3 mg/mL) was incubated with tubulin polymerization buffer in pre-warmed 96-well microtiter plates at 37°C in the presence of 0.5 nM, 5 nM, 10 nM ERI and 1 μM PTX. Then absorbance was monitored continuously for 1 h at 340 nm.

### Live cell imaging and image analysis.

RPE1 cells stably expressing Histone H2B-RFP or MDA-MB-231 cells stably expressing Histone H2B-mCherry were plated on 4-chaamber 35 mm glass bottom dish at least one day prior to do imaging (#1.5 glass, Cellvis). Cells were treated with DMSO (control), PTX (10 nM), or ERI (10nM) 1 hour prior to imaging. High-temporal live-cell imaging was performed using a Nikon Ti2 inverted microscope equipped with a Hamamatsu Fusion camera, spectra-X LED light source (Lumencor), Shiraito PureBox (TokaiHit) and a Plan Apo 20x objective (NA = 0.75) controlled by Nikon Element software and Metamorph (Molecular Devices). Cells were recorded at 37°C with 5% CO2 in a stage-top incubator using the feedback control to maintain the growing media’s temperature (Tokai Hit, STX model). Image analysis was performed using Nikon Element software. Mitotic stages were determined by nuclear staining. The mitotic duration was measured from nuclear envelope breakdown (NEBD) to anaphase onset. Incidences of multi-nuclei, mitotic slippages and unaligned chromosome were analyzed. The experiments were independently repeated 2–3 times for mitotic duration measurements (total of n = 100), and P-values between variants were calculated by One-Way Anova and two-tailed t-test. P-values < 0.05 were considered significant.

### Immunofluorescence (IF).

MM231 cell lines(DMSO, PTX-short, PTX-long, ERI-short, ERI-long) and RPE cell lines (DMSO, PTX-short, ERI-short) were plated on the cover glass of 6-well plates at approximately 5×10^4^ cells /well. After cells adhesion, the cells were treated with DMSO or PTX or ERI for 24 h. Then Cells were fixed with 4% paraformaldehyde in PBS for 15 minutes at RT, permeabilized and blocked with 2.5%FBS, 0.2M glycine and 0.1% TritonX-100/PBS overnight at 4°C. The primary antibodies were diluted in PBS with 1% BSA, and incubated 1 hour at RT. The secondary antibodies were dissolved in PBS and incubated 30 min at RT with blocking out light. Cell nuclei were stained with DAPI followed by imaging using a Zeiss AxioCam HRC microscope camera using a 60x objective lens. A DNA Damage Detection Kit was used as the protocol. Characteristic cells were independently counted in each of the 10 fields of vision for each stain and statistically compared by unpaired t test.

### Western blotting (WB).

Cell lysates were extracted with RIPA bufer (#16488–34, Nacalai) containing protein inhibitor (#A32955, Thermo Fisher). Proteins (20 μg) were resolved by SDS-PAGE using a 15% XV PANTERA Gel, and transferred to Immobilon-P PVDF membranes. After blocking with 5% skim milk for 60 min, except phosphorylated protein blocked with 5% Bovine serum albumin, the membranes were immersed with diluted primary antibody and shaked for overnight at 4°C, followed by shaking with secondary antibody for 1 hour at RT. Proteins were visualized using Chemi-Lumi One Super (#02230) or Chemi-Lumi One Ultra (#11644) which are chemiluminescent substrates. LAS4000 UV mini were used for blot detection. The lysates and the antibodies were washed by using 5% TBST buffer.

### Cell fractionation assay.

The Thermo Scientific^™^ NE-PER^™^ Nuclear and Cytoplasmic Extraction Reagents (#78833) was used for separation of cytoplasmic and nuclear extracts from MM231 cell lines (DMSO, PTX-short, PTX-long, ERI-short, ERI-long). Nuclear components and cellular components were extracted from each cells following the protocol (https://www.thermofisher.com/order/catalog/product/78833).) Then we evaluated the differences of cGAS expression between nuclear components and cellular components by WB.

### SiRNA transfection.

The cGAS specific siRNAs (Hs_C6orfl 50_8, Hs_C6orfl 50_7, Hs_C6orfl 50_6, Hs_C6orfl 50_5, QIAGEN) (5 nmol/L) and negative control siRNA (QIAGEN) (5 nmol/L) were transfected into MM231 cells by using Lipofectamine RNA iMAX Reagent (Invitrogen).

### RT-PCR.

RNA was extracted from MM231 cell lines according to the manufacture’s protocols of the RNeasy Mini Kit (QIAGEN). The RNA was reverse transcribed to cDNA using SuperScript VILO cDNA synthesis Kit and Master MixRNA (Thermo). PCR reaction solution was prepared using TB Green Fast qPCR Mix (Takara Bio) and human cGAS primers (5’-TGCAAAGGAAGGAAATGGT-3′ and 5′-TTTAAACAATCTTTCCTGCAACA-3′). PCR reactions were performed on an Applied Biosystems 7300. To evaluate the relative expression of proteins, the 2-ΔΔCT method was used to compare with control.^35^

### Proliferation assay.

At 24h after siRNA (Hs_C6orfl 50_5, negative control siRNA) transfection into MM231, the cells were detached and seeded at 5×10^4^ cells/ml (set as 0 hour). Then DMSO, PTX, and ERI were added to both MM231 cells and KD-cGAS MM231 cells. Cell proliferation was assessed by measuring absorbance at 450 nm using Cell count Reagent SF (Nacalai Tesque) and a plate reader (Bio-Rad Laboratories).

### Tripan blue assay.

The cell lysates from MM231 cell lines (DMSO, PTX-short, ERI-short, KD-cGAS-DMSO, KD-cGAS-PTX, KD-cGAS-ERI), which were 24 hours after PTX or ERI treatment, were extracted and diluted equally by tripan blue (Nacalai, #20577–34). The total number of cells and the viable cells were counted by Bio RAD TC20 Automated Cell Counter. The procedure was performed three times independently.

### Patients and samples.

All clinical samples were obtained from breast cancer patients who assigned to randomized Phase JONIE-3 clinical trial (UMIN000012817). In this multicentre randomised study, 121 patients were diagnosed with invasive breast cancer and performed neoadjuvant chemotherapy (NAC) between December 2013 and April 2016. The patients were randomly assigned to 2 different NAC groups: ERI group (eribulin followed by fluorouracil, epirubicin, and cyclophosphamide; FEC) or PTX group (paclitaxel followed by FEC). The patients of both groups were performed biopsy before and after chemotherapy. In the trial, 115 cases were analyzed for safety. We defined pCR as no invasive residual cancer in the breast. JONIE3 clinical trial protocol was approved by the institutional review board (IRB) of Tokyo Medical University on Dec. 25, 2013 (approval number: SH2588) and subsequently by all participating institutions. No tissues were procured from prisoners or vulnerable groups. Both JONIE3 clinical trial and this study were approved by the IRB at department of medicine, Chiba University (approval number: M10112) and all methods were performed in accordance with the Declaration of Helsinki and domestic relevant guidelines and regulations. Study participant names and other HIPAA identifiers are not included in all texts/figures/tables/images.

### Immunohistochemistry (IHC).

IHC was performed using tissue samples obtained by JONIE-3 clinical trial.^2^ We can evaluate 56 clinical samples in out of 115 cases. Four cases were excluded because it was insufficient for evaluation of IHC, 55 cases were excluded because we cannot permit using the samples. For IHC staining, tissue samples were thin sliced at 4 μm thickness. Antigen retrieval was performed by autoclaving for 25 min, and endogenous peroxidase activity was inactivated with 3% hydrogen peroxide. Following nonspecific protein blocking with 5% BSA except for using STING antibody blocked with 5% skim milk, the slides were stained with a cGAS or IFNβ or RAD51 antibody. The sections were incubated overnight with primary antibodies at 4°C. The sections were incubated with primary antibodies overnight at 4°C, and stained with secondary antibodies (DAKO, #k4003112) for 30 min at room temperature, followed by staining with diaminobenzidine for 5 min (Nakalai Tesque). The IHC was evaluated by H-score at hot spot.^24^ H-score was calculated by adding the percentage of positive cells multiplied by the weighted intensity of staining: H-score= (1×% weak positivity)+ (2×% medium positivity)+ (3×% strong positivity). A positive cGAS-score was defined as beyond 80, a positive STING-score was defined as beyond 120, a positive IFNβ-score was defined as beyond 180 and a positive RAD51-score was defined as beyond 100. The H-scores for each group were calculated and compared using the unpaired t-test. Simple linear regression was used to examine the correlation between the score of cGAS and IFNβ, or cGAS and RAD51.

### Statistical analysis.

Statistical analysis were performed using Graphpad Prism version 9 statistical software. In all analysis, P < 0.05 was judged statistically significant.

## Figures and Tables

**Table 1a.** Immunostaining results of cGAS, STING, IFNβ, and RAD51 in both PTX and ERI groups are shown. The results were separately classified into pCR and non pCR cases.

Antibody	agents	H score	pCR (n)	non pCR (n)	p value
cGAS	Paclitaxel	Low	3	14	0.2919
		High	4	5	
	Eribulin	Low	0	13	0.0186
		High	5	9	
STING	Paclitaxel	Low	5	10	0.8124
		High	3	9	
	Eribulin	Low	2	12	0.8614
		High	3	10	
IFNβ	Paclitaxel	Low	3	8	0.4098
		High	5	11	
	Eribulin	Low	0	14	0.0180
		High	5	8	
RAD51	Paclitaxel	Low	5	11	0.4519
		High	2	9	
	Eribulin	Low	4	8	0.1233
		High	1	13	

**Table 1b.** We evaluated the correlation between the H-score of cGAS/RAD51 and pCR in both PTX and ERI group.

Agents	cGAS/ RAD51	pCR (n)	non pCR (n)	p value (high/low vs the others)
Paclitaxel	high/ low	3	5	0.5786
	Others	5	14	
Eribulin	high/ low	4	4	0.0028
	Others	1	17	

## Data Availability

The datasets generated during and/or analysed during the current study are available from the corresponding author on reasonable request.
